# Precursor Dendritic Cell Proliferation in Multiple Myeloma: A Precursor to Acute Myeloid Leukemia

**DOI:** 10.3390/hematolrep18010003

**Published:** 2025-12-25

**Authors:** Katarina Reberšek, Saša Anžej Doma, Matevž Škerget, Helena Podgornik

**Affiliations:** 1 Department of Haematology, University Medical Centre Ljubljana, 1000 Ljubljana, Slovenia; katarina.rebersek@kclj.si (K.R.); sasa.anzej.doma@kclj.si (S.A.D.); matevz.skerget@kclj.si (M.Š.); 2 Faculty of Medicine, University of Ljubljana, 1000 Ljubljana, Slovenia; 3 Faculty of Pharmacy, University of Ljubljana, 1000 Ljubljana, Slovenia

**Keywords:** dendritic cells, multiple myeloma, acute undifferentiated leukemia

## Abstract

**Background**: Dendritic cells (DCs) are heterogeneous antigen-presenting cells that bridge innate and adaptive immunity. Recent classifications of hematolymphoid neoplasms highlight the complex origins of DC-related neoplasms. DCs have also been associated with the progression of multiple myeloma (MM). This report presents the case of a patient with MM in whom bone marrow analysis revealed an unusual additional clonal population of immature cells, in addition to plasmacytoid DCs, that later evolved into plasmacytoid dendritic cell proliferation associated with acute myeloid leukemia (pDC-AML). **Methods**: The bone marrow of a 69-year-old man with neutropenia and thrombocytopenia was examined by morphology, immunohistochemistry, flow cytometry, cytogenetics, fluorescence in situ hybridization (FISH), and next-generation sequencing (NGS). Serial assessments were performed before and during treatment with bortezomib and dexamethasone for MM, and later with daunorubicin/cytarabine for AML. **Results**: Initial bone marrow analysis revealed coexisting clonal plasma cells with t(11;14) and a population of CD34+/CD123+/CD45RA+ cells lacking lineage markers, in addition to pDCs, suggestive of precursor DCs rather than acute undifferentiated leukemia. Cytogenetic analysis identified a small clone with isolated del(20q), which corresponded in size to the clone of undifferentiated cells and to the clone with pathogenic variants detected by NGS in the *BCOR*, *RUNX1*, and *SRSF2* genes. Myeloma therapy decreased both MM and undifferentiated cells; however, within four months, pDC-AML evolved with del(20q) and higher variant allele frequencies of the previously detected gene variants. Remission was achieved with standard AML chemotherapy. **Conclusions**: This case supports evidence that MM-associated immune dysfunction and bone marrow niche alterations may promote secondary myeloid malignancies independently of cytotoxic therapy. It demonstrates the earliest events in pDC-AML evolution. Furthermore, the immature immunophenotype raises the question of appropriate treatment, since a diagnosis of acute undifferentiated leukemia can be established.

## 1. Introduction

Dendritic cells (DCs) are mononuclear cells found in blood, lymphoid organs, and tissues. They play a key role in immunosurveillance and serve as a bridge between the innate and adaptive immune responses. DCs are heterogeneous and consist of several subtypes [1,2]. Discrepancies regarding the origin of these cells are reflected in recent classifications of hematolymphoid tumors. The World Health Organization (WHO) classification places histiocytic and DC neoplasms after myeloid neoplasms, as they share common myeloid precursors from which monocytic, histiocytic, and dendritic cell lines develop. The WHO classification recognizes two types of neoplastic plasmacytoid dendritic cell proliferations: blastic plasmacytoid dendritic cell neoplasm (BPDCN) and mature plasmacytoid dendritic cell (pDC) proliferation (MPDCP) associated with myeloid neoplasms, most frequently chronic myelomonocytic leukemia (CMML), and less commonly myelodysplastic neoplasm (MDS) or acute myeloid leukemia (AML) [3,4]. In the International Consensus Classification (ICC), BPDCN is recognized as a major category within myeloid neoplasms [5] and as a separate entity from histiocytic and dendritic cell neoplasms [6].

pDCs are normally a rare immune subset (<1% of bone marrow cells) [7]. Some studies suggest that pDCs in humans arise not only from common myeloid progenitor but also from multipotent lymphoid progenitors [8]. pDC-AML is an uncommon neoplasm that shows cross-lineage antigen expression and has a poor prognosis [3]. It is characterized by a specific phenotype, clearly distinct from BPDCN: pDC-restricted markers (CD4+/CD56–/CD303+/CD304+) combined with CD34+ expression [9]. *RUNX1* mutations are found in over 70% of pDC-AML patients, which is much more frequent than in AML without pDC expansion and BPDCN [10]. Mutations in epigenetic regulators, including *ASXL1, DNMT3A*, and *TET2*, occur in 25–35% of patients, similar to the most frequent splicing factor mutations in *SRSF2* [9,10]. DCs have also been observed in other malignancies. DCs accumulate in the bone marrow during progression from monoclonal gammopathy of undetermined significance to multiple myeloma (MM) and exert immunosuppressive and tumor-promoting properties [2,8,9,10,11].

Here, we report the case of an MM patient in whom 20% undifferentiated cells were detected in the bone marrow compartment, which were reversed by dexamethasone and bortezomib, followed by subsequent progression to pDC-AML.

## 2. Case Presentation

A 69-year-old male patient was referred to our department because of neutropenia (WBC 1.17 × 10^9^/L, neutrophil granulocytes 0.44 × 10^9^/L) and mild thrombocytopenia (99 × 10^9^/L); hemoglobin was 12.5 g/dL. M-protein was not present in serum, and the light chain ratio was normal.

Cytomorphologic examination of the bone marrow stained by Pappenheim revealed two populations: 22% plasma cells and 33% immature cells, which were morphologically primitive, had a high nuclear-to-cytoplasmic ratio, and circular nuclei; however, the cytoplasm was pale.

**Figure 1:**
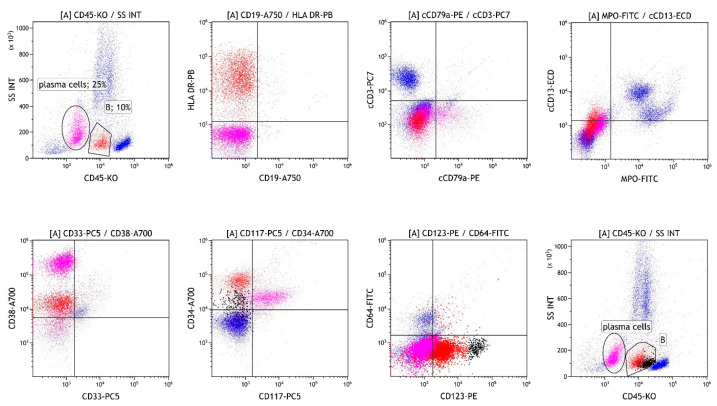
Flow cytometric analysis of the bone marrow on first analysis before treatment showing infiltration with MM cells (magenta), cells lacking common lineage markers (B, red), and pDC (B, black). MM: multiple myeloma.pDC: plasmacytoid dendritic cell..

Bone marrow biopsy confirmed the presence of two populations: 50–60% atypical plasma cells with aberrant expression of CD117 and Cyclin D1, and no light chain restriction, and 40% CD34/TdT/CD99-positive cells, which were not clearly specified and could also represent acute leukemia. The patient was asymptomatic, with normal calcium levels and renal function, and no lytic lesions or extramedullary infiltrates detected on PET-CT. Flow cytometric analysis was performed in the bone marrow sample. Briefly, the sample was subjected to erythrocyte lysis with ammonium chloride. After lysis, the cells were washed twice with phosphate-buffered saline. The cells were incubated with monoclonal antibodies (Beckman Coulter, Brea, CA, USA). After incubation, the acquisition on the flow cytometer (Navios, Beckman Coulter, Brea, CA, USA) was performed. The optical alignment and fluidic system were checked daily prior to sample acquisition using Flow-Check Pro Fluorospheres (Beckman Coulter, Brea, CA, USA) and standardisation was performed weekly using Flow-Set Pro Fluorospheres (Beckman Coulter, Brea, CA, USA) according to the manufacturer’s instructions. Analysis of the bone marrow revealed 25% plasma cells expressing an aberrant immunophenotype (CD19−/CD27−/CD28+/CD38+/CD45−/CD56+/CD81−/CD117+/CD138+/CD200+). We also detected 10% of cells lacking common lineage markers (Figure 1) but strongly expressing CD34. The diagnosis of acute undifferentiated leukemia was possible, but after further assessment of the immunophenotype (CD3−/CD14−/CD16−/CD19−/CD20−/CD34+/CD45RA+/CD56−/CD123+), infiltration of precursor DCs in the presence of MM cells was suspected. An excess of pDCs (3%) was also detected among the leukocytes by flow cytometry. The diagnosis of BPDCN was ruled out due to CD4, CD56, and TCL1 negativity.

**Figure 2:**
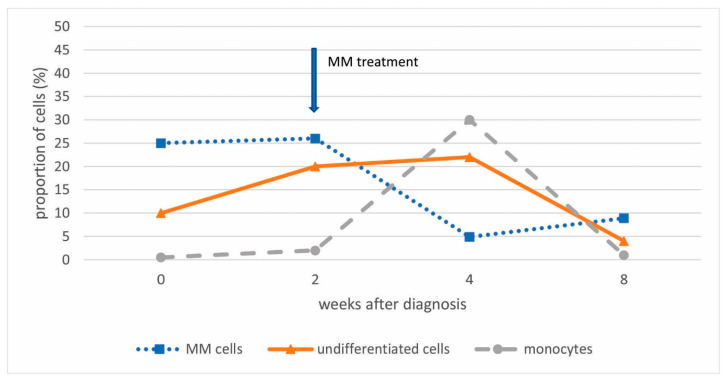
The extent of the bone marrow infiltration with MM cells, undifferentiated cells, and monocytes at diagnosis and during patient treatment.

Since acute leukemia was a differential diagnosis, a bone marrow evaluation was repeated after two weeks while awaiting the results of genetic tests. The second analysis showed an increase in cells lacking common lineage markers to 20%, with infiltration by pDCs and MM cells remaining unchanged (Figure 2).

Fluorescence in situ hybridization (FISH) analysis was performed on plasma cells isolated from bone marrow by CD138-positive immunoselection using EasySep™ Human Bone Marrow CD138 Positive Selection Kit (https://www.stemcell.com/easysep-human-bone-marrow-cd138-positive-selection-kit-ivd.html; accessed on 15 December 2025) (Stemcells, Vancuver, BC, Canada). Cytogenetic analysis was performed using a panel of DNA probes for detection of del(17p), t(11;14), chromosome 1 aberrations (1q gain/1p deletion) (all from Cytocell, Cambridge, United Kingdom) and hyperdiploidy (chromosomes 5/9/15) (Vysis, Downers Grove, IL, USA). Reporting cut-off levels were set at 20% for numerical aberrations and 10% for *IGH* translocations, chromosome 1 aberrations, and del(17p). A translocation t(11;14) with *IGH::CCND1* fusion was confirmed in 77% of the plasma cells. Del(17p), chromosome 1 aberrations (1q gain/1p deletion), and hyperdiploidy (chromosomes 5/9/15) were excluded.

Karyotyping and NGS analysis with a panel of myeloid genes were also performed on bone marrow cells. The variants were detected on isolated DNA using anchored multiplex PCR for library preparation. Selected gene regions were enriched with an amplicon-based approach using the VariantPlex Core Myeloid panel (ArcherDX, Boulder, CO, USA).

Cytogenetic analysis of the GTG banded chromosomes revealed a small clone (12%) of cells with a deletion on the long arm of chromosome 20, which was also confirmed by FISH analysis for the target region on 20q (Vysis, Downers Grove, IL, USA) with a clone of comparable size (13%). The NGS analysis revealed pathogenic variants in the genes *BCOR*, *RUNX1*, and *SRSF2* with variant allele frequencies of 13%, 7%, and 9%, respectively. No recurrent genetic aberrations defining acute leukemia were detected. The quantity of plasma cells was insufficient to perform separate NGS analyses on selected plasma cells. However, repeated FISH analysis ruled out the simultaneous presence of t(11;14) and del(20q) in the plasma cells.

The diagnosis of smoldering MM with additional undifferentiated cells was made based on a complete diagnostic workup [12]. Based on the comparable clone size, we hypothesized that the clonal aberrations corresponded to a population of undifferentiated cells. However, this was not certain, as the analysis was not restricted to these cells.

Because of the substantial bone marrow infiltration with MM and neutropenia, treatment with dexamethasone and bortezomib was initiated in order to potentially unmask the undifferentiated cell population. Repeated flow analysis confirmed a significant decrease in the MM population (8%) after only one cycle (two weeks) of therapy, as well as a decrease in undifferentiated cells (4%), with a transient increase in monocytes (week 4 after diagnosis) (Figure 2). The patient’s neutrophil count improved, and platelet levels returned to baseline after a transient decline. A watch-and wait approach was subsequently adopted.

**Figure 3:**
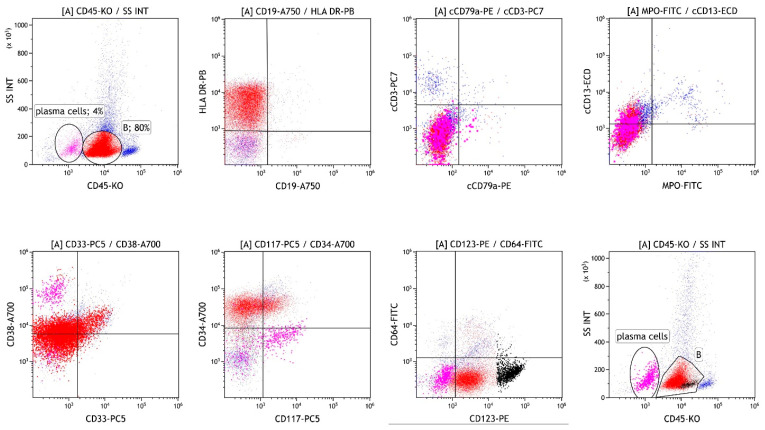
Flow cytometric analysis of the bone marrow four months after initial diagnosis, showing infiltration with MM cells (magenta), minimally differentiated myeloblasts (B, red) and pDC (B, black).

After 4 months, bone marrow diagnostics were repeated due to progressive neutropenia. Flow cytometric analysis revealed 80% of cells heterogeneously expressing CD117, CD33, and CD13, corresponding to minimally differentiated myeloblasts (Figure 3). In addition, an increase in pDCs to 7% was observed, while infiltration with MM cells decreased to 4%. A diagnosis of pDC-AML was established.

Repeated cytogenetic analysis confirmed an increase in the clone with del(20q), which comprised 75% of the metaphases. Pathogenic variants in the genes *BCOR, RUNX1*, and *SRSF2* were again detected by a second NGS analysis, with significantly higher allele frequencies of 86%, 43%, and 45%, respectively.

The patient was subsequently treated for AML with three cycles of liposomal daunorubicin and cytarabine. Although the confirmed MDS-related somatic mutations classified the AML as high risk according to ELN, complete hematological remission was achieved. An NGS analysis performed after completion of treatment was negative for all previously detected variants. The patient was assessed and deemed unsuitable for allogeneic hematopoietic stem cell transplantation; therefore, maintenance therapy with oral azacitidine has been initiated as an alternative.

In accordance with the Declaration of Helsinki, written informed consent was obtained from the patient for the use of biological material and data obtained through analysis for research purposes, while maintaining anonymity. The patient also consented to the publication of the results for scientific purposes.

## 3. Discussion

pDCs typically account for less than 1% of cells in bone marrow [10]. Their increased population can result from malignant transformation (BPDCN) or, more commonly, from an associated malignancy [13]. Our patient presented with a small clone of undifferentiated cells (10%) and pDCs (3%) accompanying the prominent MM clone. Due to the immature immunophenotype of the undifferentiated cells, acute undifferentiated leukemia could be diagnosed if infiltration exceeds 20%. However, since DCs highly express HLA class II molecules and lack common lineage markers [4,7,11], and precursor DCs highly express CD34 [1], infiltration of precursor DCs in the presence of MM cells was suspected instead. Given the pathogenesis of MM, in which multiple DC deficiencies are known, accumulation of precursor DCs seemed reasonable [7]. The diagnosis of BPDCN was also ruled out due to negativity for CD4, CD56, and TCL1 [9]. Additionally, chromosomal aberrations (del(20q)) and variants in the genes *BCOR, RUNX1*, and *SRSF2* were not diagnostically specific. However, they indicated another clonal expansion in the bone marrow, likely MDS. Considering that cytopenias could accompany MM and the relatively small size of the clone, it could also correspond to clonal hematopoiesis of unknown significance (CHIP). Infiltration with MM and undifferentiated cells was reversed by dexamethasone and bortezomib (Figure 2). However, after four months, AML was confirmed at a follow-up bone marrow aspiration performed due to progressive neutropenia. Repeated genetic analyses of bone marrow infiltrated with blasts confirmed the same genetic aberrations, including a variant in *RUNX1*, previously detected in the small clone of bone marrow cells. Expanded pDCs in pDC-AML have been shown to share the same mutational landscape as CD34+ blasts and arise in association with *RUNX1* mutations, which are the most common somatic alterations in pDC-AML, found in over 70% of patients [3,10]. Based on this, we assumed that a small clone of precursor DCs developed into full-blown pDC-AML. These results also prompt discussion regarding appropriate treatment, especially because of CD123 expression on clonal undifferentiated cells and even stronger expression on pDCs [14]. Based on the kinetics of different cell compartments during bortezomib and dexamethasone treatment, which led to an increase in monocytes, we can also infer the rationale for corticosteroid use and gain insight into cell differentiation under such treatment (Figure 2). Although it has been suggested that monocytes and DCs are distinct from each other in the steady state, potential interconversion has not been excluded [1]. The impact of AML treatment at that time could not be taken into account, as it had not yet started.

It has been shown that patients with MM or even monoclonal gammopathy of undetermined significance have a significantly higher risk of developing MDS and AML [15]. Although the presence of CHIP is one of the standard risk factors for the development of AML, CHIP expansion was found unlikely to be the mechanism underlying the increased incidence of MDS/AML among patients with MM [16]. One proposed mechanism for the frequent association of MM with MDS or AML is dysregulation of the immune system or MM-induced changes in the bone marrow niche [16]. With documented clonal evolution—spanning MM, (precursor) DCs, and ultimately pDC-AML—our case, involving two malignancies and somatic non-CHIP mutations (*RUNX1*, *BCOR*), supports evidence that MM may promote secondary myeloid malignancies independently of cytotoxic therapy. Furthermore, the immature immunophenotype of precursor DCs and their close association with pDCs and monocytes raises the question of appropriate and timely therapeutic decisions.

As increased pDCs can result from an associated malignancy [10], we assume that screening for pDCs at MM diagnosis would not be beneficial. However, an expanded population (≥10%) of CD34-positive cells lacking common lineage markers and non-CHIP mutations [16] would warrant close observation and potentially early intervention with immunomodulatory therapies that disrupt the MM bone marrow niche. A limitation of our study is that only one cycle of MM therapy was evaluated, and outcomes after additional cycles or alternative treatment strategies remain unknown; additional data are required.

## 4. Conclusions

Our case may indicate the proliferation of dendritic progenitor cells that can mimic acute undifferentiated leukemia. This underscores the importance of immunophenotyping that extends beyond the standard antibody panel used to distinguish normal from MM cells. Although DC neoplasms do not harbor a specific gene profile, detection of a variant in *RUNX1* can aid in differential diagnosis. Cytogenetics, together with molecular profiling, can further confirm the cell lineage involved. Such a comprehensive approach is essential for accurate diagnosis, risk stratification, and therapeutic decision-making in patients with overlapping hematologic neoplasms.

## Data Availability

Data cannot be shared openly but are available on request from authors.

## Abbreviations

The following abbreviations are used in this manuscript:

BPDCN	Blastic plasmacytoid dendritic cell neoplasm
DCs	Dendritic cells
FISH	Fluorescence in situ hybridization
ICC	International Consensus Classification
MM	Multiple myeloma
MPDCP	Mature plasmacytoid dendritic cell proliferation
NGS	next-generation sequencing
pDC-AML	Plasmacytoid dendritic cells proliferation associated with acute myeloid leukemia
WHO	World Health Organization
